# Supplementary material for: Managing urban solid waste in Ghana: Perspectives and experiences of municipal waste company managers and supervisors in an urban municipality
Source: PLoS One. 2021 Mar 11;16(3):e0248392. doi: 10.1371/journal.pone.0248392 (PMC7951920; doi:10.1371/journal.pone.0248392)
Supplement: S1 File — (DOC) [file pone.0248392.s001.doc]

**Managing urban solid waste in Ghana: Perspectives and experiences of municipal waste company managers and supervisors in an urban municipality**

This interview schedule is designed to elicit information on Managing urban solid waste in Ghana: Perspective and experiences of municipal waste company managers and supervisors in an urban municipality. The study is purely on academic grounds and is intended to make policy recommendations for waste management in Ghana. The study will be non-invasive and individual privacy will be respected. You are assured that all the information supplied will be treated confidentially. Kindly respond as honestly and completely as possible.

**Brief Introduction**

- Welcome the participants and appreciate them for taking time off their busy schedule to be present for the interview session
- Explain the purpose of the study
- Explain what the whole process involves such as the main topic for the discussion
- The interview shall be confidential
- Recoding of proceedings and the rationale of doing it
- Voluntary participation and having the right to stop in the course of proceedings without any consequences
- Give the participants the opportunity to ask questions
- The signing of the informed consent form by the participant
- Switch on audio-recorder

**Section I: Socio-demographic characteristics of participants**

You are requested to provide information about yourself by indicating with a cross (x) or check (√) or, where applicable, entering a number in the spaces provided. The questionnaire is anonymous, which means it will not be possible to identify you or link any responses to you.

**Gender:**

Male ( ) Female ( )

**Age:**  ( )

**Marital Status**

Single ( ) Married ( ) Divorced/separated ( ) Widow/Widower ( )

**Religion**

Christian ( ) Muslim ( ) Traditionalist ( ) No religion ( )

**Educational Level**

MSLC ( ) JSS ( ) Vocational training ( ) None ( ) SHS ( ) Tertiary ( )

**What is your employment category?**

a) Casual () b) Permanent ()

**Number of years in the workplace:**

1-5 years ( ) 6-10 years ( ) 11-15 years ( ) 16-20 years ( ) over 20 years ( )

**Table 2. Interview guide for solid waste management company managers & supervisors**

| **Q1** | **Solid waste disposal methods** | 1. **Please kindly tell me what are the normal practices of solid waste management in your company? Probe what types of solid waste do you collect?** 2. **Briefly explain the procedure for waste collection and handling of various types of solid waste from the point of collection till final disposal?** 3. **Please kindly tell me why do you use these procedures in solid waste management?** 4. **Please tell me whether there are sometimes unexpected types of solid waste? Please give me examples of unexpected waste? Please kindly tell me the procedure for collecting unexpected solid waste?** 5. **How are the present solid waste collection, handling, and disposal responsibilities defined in the job descriptions of staff involved? (Provide copies).** |
| --- | --- | --- |
| **Q 2** | **Problem of Solid Waste** | 1. **What are the problems with domestic solid waste management in the Ho Municipality? Probe if he/she says yes/no to the problems, then ask why he /she thinks there are or no problems. Please tells us your experiences and views on solid management** 2. **What are the causes of the problem of domestic solid waste management and why? What do you think can be done about domestic solid waste management in Ho Municipality?** |
| **Q3** | **Work-related problems** | 1. **Could you tell me the common work-related problems and illnesses that staff commonly experience?** 2. **What are the causes of work-related problems? Probe any problem mentioned asking for causes** 3. **Why do you think these are the causes of work-related problems? How do you think domestic waste collectors’ behavior could cause work-related problems or illness?** 4. **Please could you tell me the extent of the work-related problems? Can you explain the health risk factors that your workers are facing? Probe (psychosocial e.g. physical). Probe who are those at risk? How did you know about those at risk? Why do you think the health-related risks happened?** 5. **What type of personnel protective equipment do you provide for your staff (gloves, goggles, etc.)?Probe for types** 6. **Please could you give me an estimated cost of work-related problems? Probe for their knowledge and awareness of diseases etc.** |
| **Q4** | **Consequences & Relationship** | 1. **What do you think will be the effect of the health-related risks on the life of domestic waste collectors and your company? Probes how will you deal with health or work-related risks?** 2. **What personal information can you give me in terms of your work as a supervisor? Please kindly tell me about your relationship with your employees?** |
| **Q5** | **Safety & Health** | 1. **Briefly explain the control measures that you have put in place to prevent health risks and accidents of staff and individual domestic waste collectors?** |
| **Q6** | **Capacity building** | 1. **Please kindly tell me the kind of skills, experience, and knowledge you have in solid waste management?** 2. **What type of training and capacity building is needed by your staff?** 3. **Why do you think such training and capacity building would be required by your staff?** 4. **Please what kind of domestic waste collectors do you employ? Why this kind of domestic waste collectors? Kindly tell me about the pressure you face in performing your tasks in solid waste management** |
| **Q7** | **Wellbeing** | 1. **What does the community think of solid waste management? What do domestic waste collectors think of solid waste management?** 2. **What are some of the things that you think can be done to improve on wellbeing and safety of domestic waste collectors?** |
| **Q8** | **Stakeholders** | 1. **Who are the stakeholders involved in the solid waste management processes in Ho Municipality? Probe .what has been their contribution to the current solid waste management system?** |
| **Q9** | **Regulation & Policies** | 1. **Please kindly tell me the kind of regulations, procedures, and practices used to meet safety policies and regulations?** 2. **How do you follow laid down-regulation and rules in performing your tasks? Please what happens if the laid down procedures have not been followed?** |
| **Q10** | **Barriers & Challenges** | 1. **Please tell me the challenges or barriers you face in the procedures and practices of solid waste management? How do the challenges or barriers affect the procedures and practices of solid waste management in your work?** 2. **How do you deal with the challenges or barriers to the procedures and practices of solid waste management? Probe each challenge mentioned, how it has been dealt with.** 3. **Please could you tell me who is responsible for enforcing local solid waste management regulations and laws in the Ho municipality? How do they enforce the regulation and laws?** |
| **Q11** | **Privatization** | 1. **The government has privatized the solid waste management system in Ho Municipality. What are the various changes that have taken place over the years (5years) in the disposal of solid waste in Ho Municipality as a result of the privatization?** 2. **What do you think is a good approach to the problems of solid waste management in Ho Municipality? Probe: Is it privatization? Is it capacity building about poor management or Is it participation based on a stakeholder approach concerning those involved already in the waste scene and those that ought to be involved? , Is it cultural, etc.?** |
| **Q12** | **Attitude & perception of community** | 1. **a) What are the attitudes and perceptions of the community towards solid waste management in Ho Municipality? Probe what are the causes of the attitudes and perceptions of the community towards solid waste management in the Municipality? Probe community reaction on dumping on their land?** 2. **What is the effect of the community attitudes and perceptions towards solid waste management in the Municipality? Please tell us the sociocultural factors influencing solid waste management** 3. **Please tell me how do you deal with the attitude and perception of the community towards solid waste management in the Municipality?** |
| **Q13** | **Attitude & perception of Domestic waste collectors** | 1. **What are the attitudes and perceptions of the domestic waste collectors towards solid waste management in Ho Municipality?** 2. **Probe what are the causes of the attitudes and perceptions of the domestic waste collectors towards solid waste management in the Municipality?** 3. **What are the effects of the domestic waste collectors’ attitudes and perceptions towards solid waste management in the Municipality?** 4. **Please tell me how do you deal with the attitudes and perceptions of the domestic waste collectors towards solid waste management?** |
| **Q14** | **Financial resources** | 1. **What financial resources have been committed by your company to effect the implementation of occupational health and safety of solid waste management? Probe on budgetary allocations for dealing with these issues.** |
| **Q15** | **Technical resources** | 1. **What technical resources have been committed by your company to effect the implementation of occupational health and safety of solid waste management? Probe manpower, kinds/level of foreign expertise desired, and challenges in attracting needed technical resources needed.** |
| **Q16** | **Motivation/ Reward** | 1. **What happens if domestic waste collectors fail to report to work or late to work?** 2. **Has the company instituted any award/reward program? Probe for types of award. Why the award? What do you think of the award?** |
|  | **Suggestion/Way forward** |  |
