# Supplementary material for: Managing urban solid waste in Ghana: Perspectives and experiences of municipal waste company managers and supervisors in an urban municipality
Source: PLoS One. 2021 Mar 11;16(3):e0248392. doi: 10.1371/journal.pone.0248392 (PMC7951920; doi:10.1371/journal.pone.0248392)
Supplement: Attachment — Submitted filename: Response to Reviewers.docx [file pone.0248392.s002.docx]

**Detailed revision notes on the revised manuscript**

**Managing urban solid waste in Ghana: Perspective and experiences of municipal waste company managers and supervisors in an urban municipality” { Manuscript ID: PONE-D-20-33283}**

We thank the two reviewers for their useful feedback and comments on the manuscript. As authors, we feel that the review comments received have helped to improve the manuscript’s quality and readability. In this section, we provide a point-by-point response on the review comments.

Reviewer **#**1

**Reviewer #1**: **Comment 1** Abstract
The study needs to state the number of possible waste management agencies (sample frame) from which the sample was taken for readers to ascertain if the sample size of two agencies are representative enough to warrant a conclusive results.

Clarity should be given on how it is different from existing studies on perspectives of waste management from managers/supervisors to avoid repetition of existing knowledge.

**Authors’ Response**

**On the comment regarding sample frame, the number of possible waste management agencies from which the sample was taken and its representativeness are provided on page 6, lines 120-124, and also on page 8, lines 163-170 of the revised manuscript.**

**On page 20, lines 406-423 of the revised manuscript, the authors have provided further and detailed reasons that explain how this manuscript and the perspectives of managers/supervisors differ and is unique from other previous studies in Ghana.**

**Reviewer #1**: **Comment 2**
Introduction
The reason provided by authors on why only waste managers/supervisors were sampled for this study is very weak and needs to be strengthened to fit purpose. Otherwise, views from waste generators in the communities could be also be obtained to corroborate the information provided by the managers/supervisors.

**Authors’ Response**

**In the revised manuscript (page 4-5, lines 87-97), the authors have provided reasons why managers/supervisors were sampled for this study. This study was designed to address specific research gaps in relation to company managers/supervisors' contribution to solid waste management in Ghana. No data was collected at the community level among waste generators.**

**Reviewer #1**: **Comment 3**
Materials and Methods
Authors should justify why only two companies were selected for the study, and by provide the calculations they used to obtain 35 respondents whose views provide a conclusive argument for the results. Even though limitations have been provided by the authors as possible bias been introduced, it still does not provide enough backing to justify that the views of 35 respondents may not be enough to provide basis for bringing about multidimensional and multilevel interventions that the authors suggest. The results therefore remain inconclusive and skewed towards the two companies which may be infinitesimal in the business of waste management in the study area, unless proper evidence based justification is provided.

**Authors’ Response**

**The justification for which only two waste companies were selected has been addressed. See page 6, lines 120-124, and also page 8, lines 163-170 of the revised manuscript**

Also, the pre-testing of questionnaires - line 160 - could/should have been done outside the two chosen companies to avoid introduction of errors/biases in ideas for the main questionnaires. Preferably outside the jurisdiction of the study area to make it more reliable.

**Authors’ Response**

**This has been addressed on page 26 lines 547-550 understudy limitations.**

**Reviewer #1**: **Comment 4**
Results/Discussion
Findings from the study present no new knowledge of what is already known as the challenges of waste management, especially in developing countries. Results should be tailored towards unearthing exciting novel approach on representing the perspectives on waste management that could trigger a response from policy makers other than the routine knowledge that yields same ineffective results.

**Authors’ Response**

**This study has unearthed context-specific perspectives of how managers/supervisors view the societal challenge of solid was management in Ghana. The findings add to existing evidence on the subject of waste management and the institutional and societal bottlenecks associated with waste management in Ghana. In the revised manuscript (page 5-6 lines 107-115), the authors have provided key results that this study unearthed and how these findings can shape the course of waste management policies in Ghana.**

**Reviewer #1: Comment 5**

General comments

1. Authors should be consistent in the use of percent and % - refer to lines 58 and 66

**Authors’ Response**

**The text has been revised to ensure consistency in the use of percent and % ( see page 3 line 65) as recommended**.

2. The study is relevant in the field of waste management. However, findings do not differ so much from existing knowledge and makes it just a repetition without bringing out any exciting information in the field.

**Authors’ Response**

**See response to comment 1 above on the added value of the study on page 20 lines 406-423**.

Reviewer **#**2

Reviewer #2; Comment: The study was very well conducted, as demonstrated in the very detailed methodology, which outline the steps taking in arriving at the said results.

The study is pure qualitative and the author outlined in detail the process of sampling, data collection, transcription and analysis, all of which was performed systematically and through a very rigorous process.

The author has also indicated that the data is available upon reasonable request from the first author.

The document is very readable and very well written. No spelling errors identified. And no grammatical errors also observed.

**Authors’ Response**

**We thank the reviewer for the feedback and comments on the manuscript. The authors are very grateful for these positive comments.**
